# Small molecule inhibition of Axl receptor tyrosine kinase potently suppresses multiple malignant properties of glioma cells

**DOI:** 10.18632/oncotarget.3952

**Published:** 2015-04-29

**Authors:** Mikaella Vouri, Qian An, Matthew Birt, Geoffrey J. Pilkington, Sassan Hafizi

**Affiliations:** ^1^ Institute of Biomedical and Biomolecular Science, School of Pharmacy and Biomedical Sciences, University of Portsmouth, Portsmouth, UK

**Keywords:** Axl receptor tyrosine kinase, small molecule inhibitor, glioma, invasion

## Abstract

Glioblastoma multiforme (GBM) often features a combination of tumour suppressor gene inactivation and multiple oncogene overactivation. The Axl receptor tyrosine kinase is found overexpressed in GBM and thought to contribute to invasiveness, chemoresistance and poor survival. Here, we have evaluated the effect of BGB324, a clinical candidate Axl-specific small molecule inhibitor, on the invasive behaviour of human GBM cells *in vitro*, as an indicator of its potential in GBM therapy and also to elucidate the role of Axl in GBM pathogenesis.

Two cultured adult GBM cell lines, SNB-19 and UP007, were treated with Gas6 and/or BGB324, and analysed in assays for survival, 3D colony growth, motility, migration and invasion. Western blot was used to detect protein expression and signal protein phosphorylation. In both cell lines, BGB324 inhibited specifically phosphorylation of Axl as well as Akt kinase further downstream. BGB324 also inhibited survival and proliferation of both cell lines in a concentration-dependent manner, as well as completely suppressing migration and invasion. Furthermore, our results indicate co-operative activation between the Axl and Tyro3 receptors, as well as ligand-independent Axl signalling, to take place in GBM cells. In conclusion, small molecule inhibitor-led targeting of Axl may be a promising therapy for GBM progression.

## INTRODUCTION

The TAMs (Tyro3, Axl, MerTK) are a subfamily of receptor tyrosine kinases (RTKs) that share structural homology in their extracellular regions, comprising a pair of immunoglobulin-like domains followed by two fibronectin type III repeats. This is followed by the RTK family-wide possession of an intracellular tyrosine kinase domain. The natural ligands for the TAMs are the vitamin K-dependent proteins Gas6 (for all three receptors, with highest affinity for Axl) and Protein S (Tyro3 and MerTK only) [1-5].

Axl has been shown to mediate cell survival, proliferation, migration and adhesion [6], whilst all three receptors have been shown to play a role in immune cell differentiation and phagocytic clearance of apoptotic cells [4, 7-9]. In addition, Tyro3 has been shown to exert neuro-protective functions in the central nervous system (CNS), where it is the most prominently expressed TAM [10, 11]. Additionally, Gas6, Protein S and Axl have been shown to be expressed by stem cells in the subventricular zone and to regulate their proliferation, survival and differentiation [12, 13]. Axl/Gas6 signalling also protects axon integrity [14]. This variety of roles indicates diversity in the signalling pathways activated by the TAMs in normal cellular homeostasis.

Aberrant TAM signalling through e.g. overexpression has been observed in multiple cancers, including gliomas [6]. Gliomas are the most common form of brain cancer and are classified morphologically according to the glial cell type from which they are derived. In addition to the morphological classification, they are usually distinguished by the WHO grading system [15]. Lower grade tumours (grade I and grade II) are well differentiated and resemble normal tissue, whereas higher grade tumours (grade III and grade IV) are anaplastic and display high cell atypia and mitotic activity [15]. Glioblastoma multiforme (GBM) is a grade IV glioma and is highly heterogeneous and invasive in nature. The current standard treatment for gliomas consists of surgical tumour resection, radiotherapy and adjuvant temozolomide chemotherapy [16, 17]. The 5-year survival rate for GBM patients is less than 5%, indicating the slow progress in therapy development and therefore the need for more effective treatments [18]. Recent studies have demonstrated overexpression of MerTK or Axl in astrocytic tumours of all grades, and co-expression of MerTK and Axl in all high grade glioma tissue samples, relative to little or no expression in normal CNS [19, 20]. Experimental blockade of Axl signalling in cultured GBM cells resulted in decreased growth and invasive potential [21]. Axl expression, and resulting invasiveness of glioma cells, has been shown to be directly regulated by the transcriptional regulator EZH2 through a mechanism independent of histone methylation [22]. Additionally, glioma cells with elevated MerTK activation showed greater resistance to pro-apoptotic stimuli as well as increased invasive potential compared to cells with MerTK expression silenced [23, 24]. Together, these observations suggest the TAMs to be an attractive new target for GBM therapy.

Novel, selective small molecule inhibitors (SMIs) of RTKs are increasingly being developed as promising new targeted molecular therapies for a variety of cancers. BGB324 (also known as R428) [(1-(6,7-dihydro-5*H*-benzo[6,7]cyclohepta[1,2-*c*]pyridazin-3-yl)-*N*3-((7-pyrrolidin-1-yl)-6,7,8,9-tetrahydro-5H-benzo[7]annulene-2-yl)-1*H*-1,2,4-triazole-3,5-diamine)] is a highly selective SMI of Axl (out of 133 tyrosine and serine/threonine kinases tested) [25]. Treatment of mesenchymal carcinoma cells with BGB324 abolished cell invasion and increased chemosensitivity [26], whilst it nullified the tumorigenic effect of triple negative breast cancer as well as blocking their acquired resistance to erlotinib (anti-EGFR SMI). BGB324 is currently in Phase 1b clinical trials for acute myeloid leukaemia and non-small cell lung cancer, and has so far been shown to be safely tolerated in clinical safety studies in healthy volunteers at doses up to 1.5 g daily (http://www.bergenbio.com/BGB324). Additionally, in breast cancer mouse models, twice daily dosing of 25, 50, and 100 mg/kg BGB324 resulted in mouse plasma concentrations of 2.4, 6.8, and 9.0 μM BGB324 respectively [25].

Here, we report our observations from a comprehensive study of the cell biological and signalling effects of BGB324 on two distinct patient-derived GBM cell lines. We demonstrate that as an SMI, BGB324 selectively inhibits Axl-mediated growth, motility, migration and invasion of GBM cells, as effectively as when Axl is genetically knocked down. Therefore, this study reveals Axl as an important mediator of gliomagenesis, and indicates that specific targeting of Axl with a highly selective SMI represents a new and promising therapeutic avenue for GBM patients.

## RESULTS

### The TAMs are overexpressed in GBM cells

**Figure 1:**
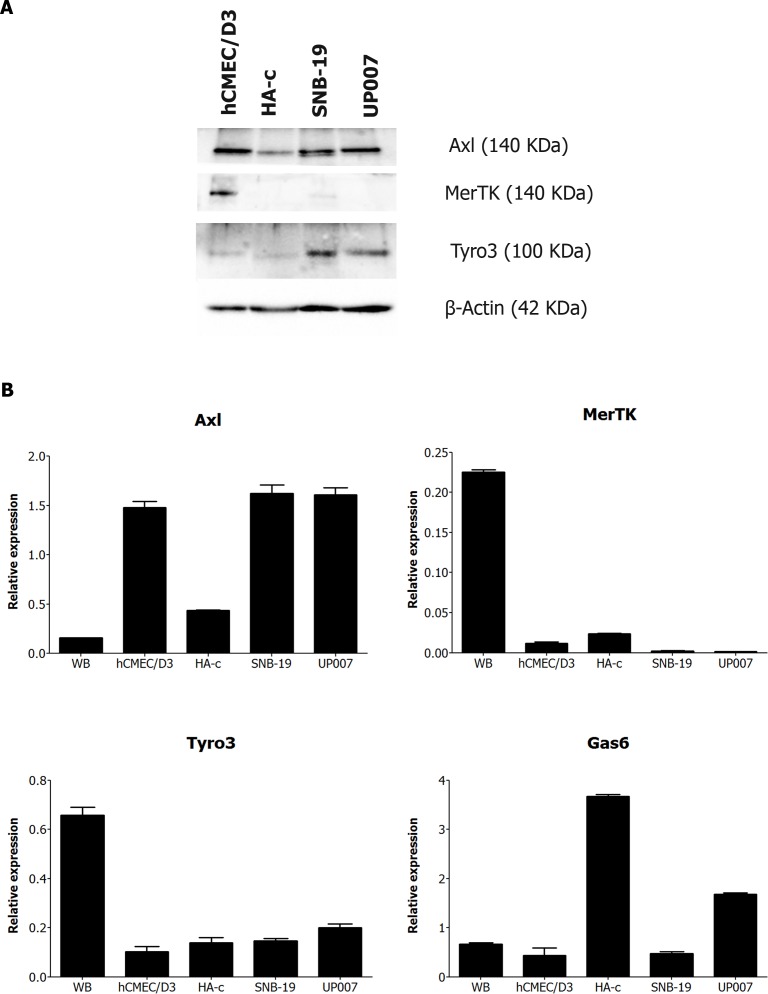
**A.** Western blot screen of TAM receptors in protein extracts from a panel of human brain cell cultures: microvascular endothelial cells (hCMEC/D3), astrocytes (HA-c) and two GBM cell lines, SNB-19 and UP007. **B.** Quantitative PCR analysis of mRNA expression of the genes for Axl, MerTK, Tyro3 and Gas6 in extracts from human whole brain (WB), endothelial cells, astrocytes, SNB-19 and UP007 cells.

We first conducted an expression screen of all TAMs in different human brain cell types, as well as the GBM cell lines involved in this study (Figure 1). In terms of protein expression, Axl and MerTK were the more prominently expressed TAMs in human brain microvascular endothelial cells (hCMEC/D3), while only Axl and Tyro3 were expressed in human cerebellar astrocytes (HA-c) (Figure 1A). Both GBM cell lines SNB-19 and UP007 showed strong expression of Axl and Tyro3 but negligible expression of MerTK. Quantitative real-time PCR analysis also confirmed our western blot results with similar expression patterns for each TAM gene (Figure 1B). Interestingly, both MerTK and Tyro3 showed high expression in whole normal human brain relative to the individual cell types, WHILST the opposite was true for Axl. Additionally, Gas6 mRNA was found to be most prominently expressed in normal human astrocytes and the UP007 cell line. Furthermore, from immunohistochemical staining for Axl in a GBM tissue array, we detected aberrant expression of Axl protein in a subset of GBM tumours (Supplementary Figure 1); this is in keeping with gene expression profiling studies showing Axl upregulation in brain tumours [27].

### Gas6 activates Axl signalling in GBM cells

**Figure 2:**
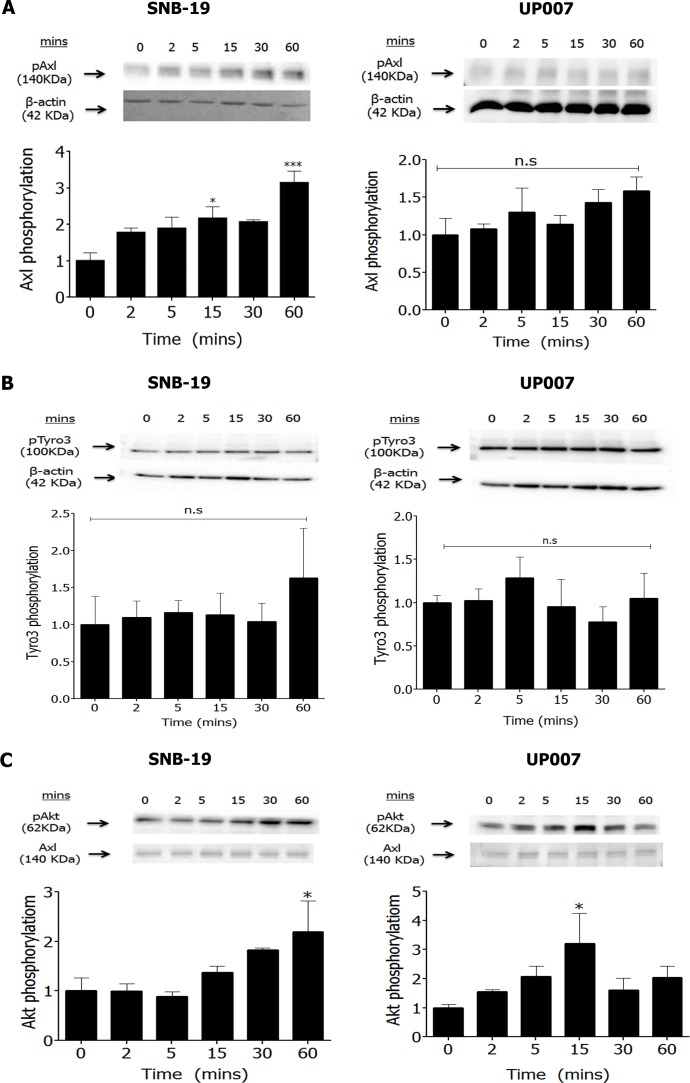
Effect of Gas6 stimulation on TAM phosphorylation and signalling in GBM cells Western blot showing time-course of Axl phosphorylation by Gas6 (400 ng/ml) in SNB-19 and UP007 cells (**A.** *n* = 5 blots for both cell lines), of Tyro3 phosphorylation in SNB-19 and UP007 cells (**B.** *n* = 3 blots for both cell lines), and of pAkt levels **C.** in SNB-19 (*n* = 3 blots) and UP007 (*n* = 4 blots) cells. Data are mean±SEM protein expression normalised against loading control protein; *p < 0.05 and ns, not significant, versus time 0.

Gas6 is the principal ligand for all three TAM receptors and has also been found to be overexpressed in glioma [20]. Exogenous Gas6 stimulated phosphorylation of Axl in SNB-19 cells but failed to do the same in UP007 cells (Figure 2A and Supplementary Figure 2A). In addition, Gas6 did not stimulate Tyro3 phosphorylation in either cell line (Figure 2B and Supplementary Figure 2B). However, Gas6 rapidly induced downstream intracellular Akt phosphorylation in both cell lines by approximately two-fold (Figures 2C and Supplementary Figure 2C).

### BGB324 selectively inhibits Axl activity and downstream signalling in GBM cells

**Figure 3:**
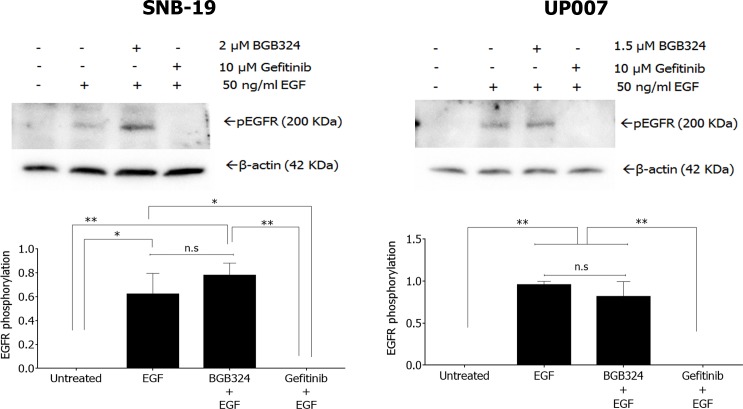
Western blot of EGFR phosphorylation by EGF in SNB-19 and UP007 cells, and influence of gefitinib and BGB324 on this Data are mean±SEM (*n* = 3 separate experiments);**p < 0.01, *p < 0.05, ns, not significant, for comparisons indicated.

BGB324 is an Axl-selective SMI, with a 15-fold greater inhibitory efficacy versus the other TAMs in biochemical assays [25]. In order to initially evaluate the specificity of BGB324, we tested its effect on the activation of EGFR in the two GBM cell lines. EGFR in cells was rapidly activated by EGF stimulation within 15min, an effect that was abolished by the EGFR-specific SMI gefitinib (Figure 3). However, pre-incubation with BGB324 had no effect on the EGFR activation by EGF (Figure 3).

**Figure 4:**
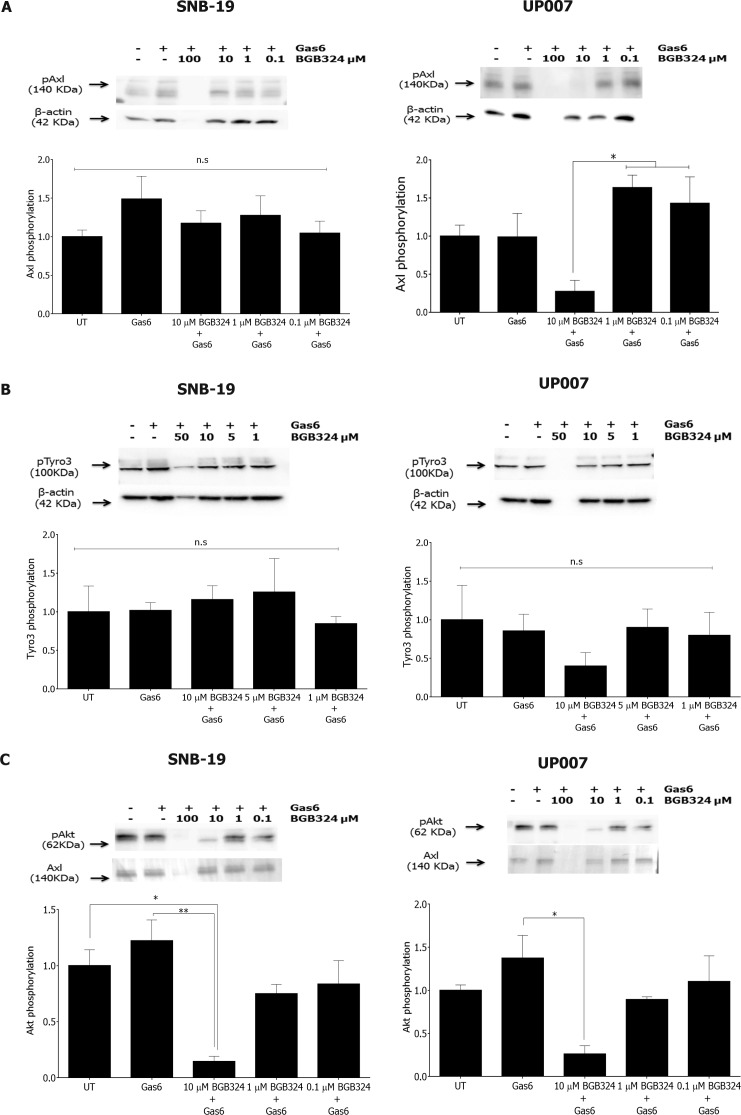
Comparative efficacies of BGB324 for inhibition of Axl, Tyro3 and Akt phosphorylation in GBM cells Western blots showing inhibition by BGB324 (0.1–100 μM) of phosphorylation of Axl **A**, Tyro3 **B.** and Akt kinase downstream **C.** stimulated by Gas6 (400ng/ml) in SNB-19 and UP007 cells. Data are mean ±SEM (n = 3 separate experiments);**p < 0.01, *p < 0.05, ns, not significant, for comparisons indicated.

We then evaluated the effect of BGB324 on Gas6-stimulated activity of Axl and Tyro3 in the GBM cell lines. Pre-incubation with BGB324 did not inhibit Gas6-induced Axl phosphorylation in SNB-19 cells at up to 10 μM. However, in UP007 cells, BGB324 markedly inhibited Axl phosphorylation (which was Gas6-independent) at 10 μM to below baseline levels (Figure 4A and Supplementary Figure 3A). BGB324 had no effect on Tyro3 phosphorylation levels in SNB-19 cells, whereas it showed a trend towards significance in inhibition in UP007 cells (Figure 4B and Supplementary Figure 3B). At 100 μM, BGB324 was toxic to all cells, as reflected by the absence of a band for β-actin in the western blots.

Next, the effect of BGB324 on activation of downstream signalling was investigated in the GBM cells. BGB324 pre-treatment significantly inhibited Akt phosphorylation in a concentration-dependent manner in both cell lines, independently of Gas6 stimulation (Figure 4C and Supplementary Figure 3C). Therefore, the Akt signalling pathway appears to emanate from Axl activation in both GBM cell lines. In contrast to Akt signalling, BGB324 had no effect on NF-κB pathway activation in both GBM cell lines (data not shown), indicating a lack of involvement of this pathway in Axl signalling in the GBM cells.

### BGB324 inhibits GBM cell growth and colony formation

**Figure 5:**
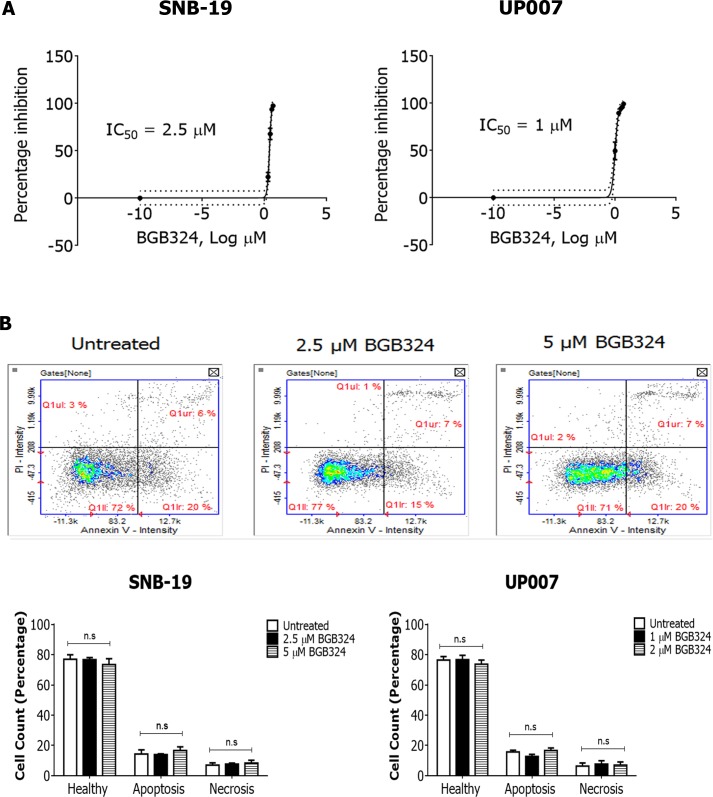
Comparative efficacies of BGB324 for inhibition of GBM cell growth and effect on cell viability **A.** MTS assay showing concentration-response effect of BGB324 on growth/viability of SNB-19 and UP007 cells. IC_50_ values were calculated from % inhibition *vs* log BGB324 concentration curves. B. Representative propidium iodide (PI) *vs* Annexin V fluorescence intensity in SNB-19 cells treated with vehicle, 2.5 μM and 5 μM BGB324. Quadrants and markers in the displayed plots were used to demarcate the various cell populations (top panel). Quantified, comparative cell counts (%) are shown for healthy, apoptotic and necrotic cells following 24h BGB324 treatment of SNB-19 and UP007 cells (bottom panel). Data are mean±SEM (*n* = 3 separate experiments); ns, not significant versus untreated.

**Figure 6:**
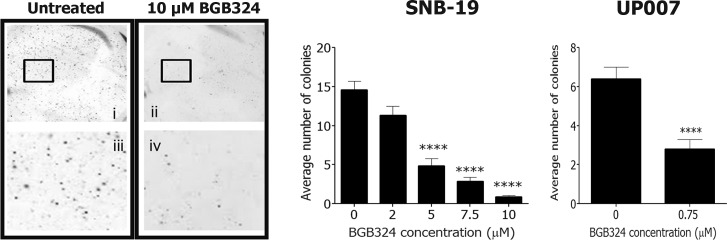
Effect of BGB324 on long-term growth of GBM cell colonies in 3D Representative micrographs of SNB-19 colonies in soft agar at 1.5x magnification (above; i and ii) and 15x magnification (below; iii and iv) untreated (left) or treated with 10 μM BGB324 (right). Histograms show average colony counts of 5 different fields per treatment for SNB-19 and UP007. Data are mean ±SEM (*n* = 3 separate experiments); ****p < 0.0001 versus untreated.

Having observed that BGB324 robustly inhibited Axl signalling in the GBM cells, we investigated the effect of BGB324 on cell growth and survival using both short-term and long-term cell-based assays. In short-term experiments (MTS assay after 72h), BGB324 significantly reduced viable cell number in both GBM cell lines in a concentration-dependent manner, with a slightly greater potency in UP007 cells (IC_50_ 1 μM) compared to SNB-19 cells (IC_50_ 2.5 μM) (Figure 5A). Additionally, no significant increase in the number of apoptotic or necrotic cells was observed in both cell lines following 24h treatment with IC_50_ and double-IC_50_ concentrations of BGB324 (Figure 5B). In long-term cell growth experiments in 3D (soft agar assays), BGB324 significantly reduced colony formation at 0.75 μM in UP007 cells, and inhibition of SNB-19 colony formation was apparent at 2 μM of BGB324, with complete inhibition of colony growth without cell death achieved at 10 μM BGB324 (Figure 6).

**Figure 7:**
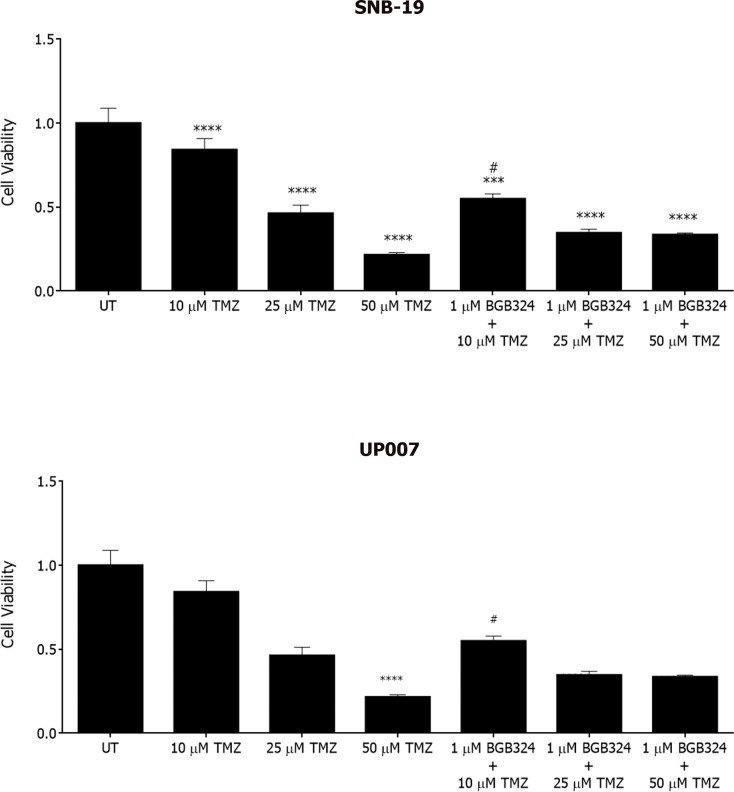
Effect of BGB324 on GBM cell growth in combination with Temozolomide (TMZ) MTS assay showing cell growth/survival of SNB-19 and UP007 cells treated with varying concentrations of TMZ alone or in combination with BGB324. Data are mean±SEM (*n* = 3 separate experiments); ****p < 0.0001, ***p < 0.001 versus untreated (UT). # indicates significance when compared to 10 μM of TMZ.

Temozolomide (TMZ) is a chemotherapeutic adjuvant to radiotherapy and is currently the only mildly successful chemotherapeutic agent for GBM, extending median survival by 3-4 months [16]. Therefore, we investigated a possible combinatorial effect of BGB324 and TMZ in GBM cells. Treatment with TMZ resulted in concentration-dependent decreases in viability of both UP007 and SNB-19 cells (Figure 7). In addition, the inhibitory effect of BGB324 at 1 μM was significantly enhanced by co-incubation with 10 μM of TMZ, versus either agent alone. Also, combinations at 50 μM TMZ did not show a significantly increased effect in the presence of BGB324, possibly due to the increased toxicity of TMZ at such a high concentration. However, all concentrations of BGB324 exhibited enhanced inhibitory effects when combined with sub-toxic doses 10 μM and 25 μM of TMZ.

### BGB324 inhibits cell migration, motility and invasion in GBM cells

**Figure 8:**
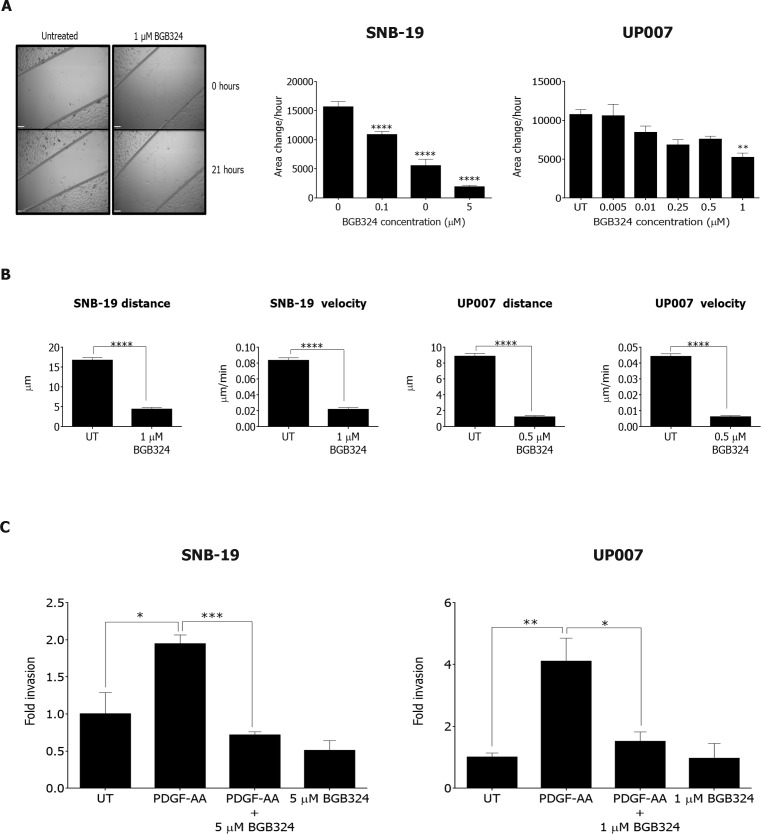
Effect of BGB324 on GBM cell migration, motility and matrix invasion **A.** Representative images from scratch wound assay for SNB-19 cells at 0 and 21 h (scale bar = 390 μm) followed by graphical representation of migration rate (area change per h) for SNB-19 and UP007 cells treated with varying concentrations of BGB324. **B.** Cell tracking experiments yielded data for average total distance travelled and average velocity for SNB-19 and UP007 cells following treatment with BGB324. **C.** Invasion assay for SNB-19 (*n* = 3) and UP007 (*n* = 4) cells following treatment with BGB324 using PDGF-AA as chemoattractant. Data are mean ±SEM (*n* = 3 independent experiments); ****p < 0.0001, ***p < 0.001, **p < 0.01, *p < 0.05 compared to untreated (UT) or as indicated.

One of the biggest challenges in GBM treatment is the highly invasive nature of the tumours, which leads to multiple relapses. We therefore tested the effectiveness of BGB324 against the migration, motility and invasive capacity of the GBM cells. BGB324 inhibited cell migration in both SNB-19 and UP007 cells in a concentration-dependent manner (Figure 8A). Moreover, cell tracking experiments revealed that sub-IC_50_ doses of BGB324 profoundly inhibited the motility of cells in both lines, affecting both average velocity of movement and total distance travelled (Figure 8B). This indicates that BGB324 can effectively block cell motility at doses below those that can cause cell death. Furthermore, Axl inhibition with BGB324 abolished invasion by both GBM cell lines through extracellular matrix that was stimulated with the glioma cell chemoattractant PDGF-AA (Figure 8C). Therefore, BGB324 inhibits migration, motility and invasion of glioblastoma cells *in vitro*, which all contribute to the aggressive clinical features of GBM.

## DISCUSSION

GBM is the most common type of primary malignant brain tumour in adults and is highly heterogeneous and aggressive, with most patients facing only a year of survival [28]. It is this heterogeneous and invasive character to GBM that renders conventional treatments ineffective. Despite intense investigation in the last decade, our understanding of the pathogenesis of these tumours still remains limited. It is thus of great importance to find new targeted therapies that will increase survival rates and quality of life for cancer patients. The TAMs have recently been associated with glioma pathophysiology, and therefore have emerged as attractive novel therapeutic targets for brain tumours as part of individualised treatment approaches. Therefore, SMIs such as the highly selective Axl inhibitor BGB324 could be more effective than the currently available treatments. The present study is the first to assess the effectiveness of an Axl-specific SMI on human GBM cells and on two cell lines with different characteristics.

In agreement with previous studies, we have shown that Axl is overexpressed in both GBM cell lines used in this study [20], relative to its normal expression level in human astrocytes. Moreover, we also observed an overexpression of Tyro3 in both GBM cell lines, whereas MerTK was found to be expressed clearly only in brain microvascular endothelial cells. This expression profile of the TAMs is consistent with previous studies [6]. In particular, the observation that, in normal tissue, Tyro3 is prominently expressed in the brain but not in astrocytes or endothelium by exclusion suggests its expression either in neuronal cells or other glial cell types, or both.

Gas6 is the common ligand for the TAM receptors, with *in vitro* studies demonstrating a higher affinity for Axl and Tyro3 than MerTK [29]. Using two GBM cell lines with a basal level of Axl activity, we observed that Gas6 activates Axl and Axl-associated signalling in SNB-19 cells but also that Axl signalling appeared to be Gas6-independent in the UP007 cell line. Gas6 did not stimulate Tyro3 phosphorylation above the basal levels apparent in either cell line. However, as MerTK was not investigated in this study, and Gas6 stimulated Akt and cell invasion in both cell lines, which cannot be explained by activation of Axl or Tyro3 in the UP007 cell line, these observations indicate a different route of stimulation by Gas6 in the GBM cells. It is conceivable however that Gas6 overexpression in GBM [20] can stimulate other TAMs in a paracrine manner or instead to supress an anti-tumour immune response. Furthermore, inhibition of Akt was observed in both cell lines following treatment with BGB324 even in the presence of Gas6, indicating that BGB324 is effective in inhibiting basal Axl activity and downstream signalling in a dose-dependent manner, irrespective of the ligand.

We investigated the effect of Axl inhibition with BGB324 on short-term growth/survival of GBM cells as well as long-term colony formation. BGB324 inhibited cell growth in both assays potently and in a concentration-dependent manner. The two cell lines exhibited different responses, with the UP007 cell line being more susceptible to inhibition (IC_50_= 1 μM) than the SNB-19 cell line (IC_50_= 2.5 μM). The slightly greater resistance of SNB-19 cells to BGB324 could possibly be due to a slight but sufficient expression of MerTK in that cell line, which UP007 completely lack, to compensate for inhibition of Axl. We probed the particular functional effect of BGB324 on cell survival by apoptosis assays, and observed that treatment of cells with BGB324 did not significantly induce apoptosis or necrosis, thus demonstrating a clear retardation in cell growth at non-toxic concentrations. Therefore, this supports the potential of BGB324 as a targeted chemotherapeutic compound, in contrast to traditional chemotherapies. Furthermore, during cell tracking experiments, a hindrance of cell division was observed under the microscope in cells treated with BGB324, although the precise effect of the drug on the cell cycle requires investigation.

Activation of the serine/threonine kinase Akt was also blocked by BGB324 in both cell lines, even in the presence of Gas6. In accordance with previous data from a study using a dominant negative form of Axl in GBM cell lines [21], blocking Axl signalling with BGB324, in addition to inhibiting cell proliferation/survival, also reduced cell migration in a dose-dependent manner. The total distance travelled and the velocity of migration were reduced significantly at sub-IC_50_ concentrations for SNB-19 and at the IC_50_ concentration for UP007 cells. Invasion was also reduced to basal levels after incubation with BGB324 following PDGF-AA stimulation in both cell lines, indicating that Axl mediates the invasive character of GBM cells in response to diverse humoral cues. Indeed, studies have shown that Axl mediates tumour invasion and chemoresistance through the activation of the PI3K/Akt pathway in breast cancer [30, 31] as well as ovarian cancer [32]. The BGB324 inhibition of GBM cell migration and invasion we observed could be the result of suppressed Akt signalling, although this requires further investigation.

The TAM family members have been demonstrated to crosstalk with other receptors such as EGFR. Axl has been shown to increase EGFR-conferred chemoresistance in lung cancer [33], while it diversifies EGFR signalling and increased resistance to targeted therapy in triple negative breast cancer [34]. In our study we observed that inhibiting Axl activation by BGB324 did not affect activation of EGFR, supporting the specificity of BGB324 for Axl as well as excluding the possibility of EGFR transactivation via Axl signalling. Moreover, neither did Gas6 stimulate further activation of EGFR.

The possibility also exists for TAM family members to heterodimerise with each other. Previous studies have shown BGB324 to be more than 100-fold more selective for Axl than Tyro3 for kinase inhibition *in vitro*. In our study, BGB324 treatment of SNB-19 and UP007 cells resulted in a reduction of Tyro3 phosphorylation in UP007 cells but not in SNB-19 cells, indicating a possible heterodimerisation between Axl and Tyro3 to occur in UP007 cells. This could also account for the different sensitivities of the cell lines towards Gas6 stimulation and treatment with BGB324, and such a phenomenon warrants further investigation.

The current treatment for GBM comprises surgical tumour resection followed by radiotherapy with adjuvant TMZ treatment, TMZ being an alkylating agent which acts by interfering with DNA replication, thus enhancing tumour cell death upon exposure to radiotherapy [17]. The ability of TMZ to cross the blood-brain barrier has made it a standard therapy for GBM, although patient survival is increased by only a few months. In recent years, the focus of TMZ combination with novel or current molecular agents has been in the spotlight [17]. In the present study, we have observed a concerted interaction between TMZ and BGB324 in terms of their anti-growth effects on GBM cells. As the mechanisms for this interaction are not clear, a study with a larger number of concentration combinations is needed to explore this further.

In conclusion, our data indicate that targeting Axl for inhibition by a specific SMI such as BGB324 has robust anti-tumour effects on GBM cells and could potentially be effective in restraining tumour invasion *in vivo* and, in combination with other agents, could effectively prolong glioma patient survival. Future studies will be needed to explore the anti-GBM tumour efficacy of Axl-selective SMIs such as BGB324, which has been established to be safe clinically and therefore may also be effective for combating highly malignant brain tumours that are driven by Axl.

## MATERIALS AND METHODS

### Cell culture

The human GBM cell lines used in this study were UP007 (established in-house from GBM biopsy resected at King's College Hospital, London under ethics permission LREC00-173/11/SC/0048) and SNB-19 (DSMZ German Brain Tumour Bank); both cell lines were authenticated in-house as described previously [35] and were mycoplasma tested. Cells were normally cultured in complete medium, comprising Dulbecco's Modified Eagle Medium (Fisher Scientific, Loughborough, UK) supplemented with 10% foetal bovine serum (Lonza, Slough, UK), 2 mM L-glutamine (Life Technologies, Paisley, UK) and 1% penicillin/streptomycin (Fisher Scientific). Cells were routinely grown to confluence in a humidified incubator with 5% CO_2_ at 37°C and passaged through dissociation with trypsin/EDTA (Lonza). Cells were always grown to near confluence before experimental use.

### Cell treatments

The SNB-19 and UP007 cells were first serum-starved for 24h, then incubated with BGB324 (gift of BergenBio, Bergen, Norway) at the concentrations and times indicated, in the absence or presence of recombinant human Gas6 protein (400 ng/ml) (R&D Systems, Abingdon, UK). In experiments using recombinant human epidermal growth factor (EGF; R&D Systems), cells were incubated with 50 ng/ml of EGF for 15min to cause activation of EGFR, whilst in inhibition experiments, cells were pre-incubated with 10 μM gefitinib (EGFR SMI; Santa Cruz Biotechnology, Santa Cruz, CA) for 2h prior to EGF stimulation. In experiments using TMZ, cells were incubated with 10 μM, 25 μM and 50 μM alone or in combination with BGB324 for 3 days.

### RNA extraction and quantitative real-time polymerase chain reaction

Cellular total RNA was isolated using GeneJET RNA purification kit (Life Technologies) according to the manufacturer's protocol. First-strand cDNA was synthesized using the nanoScript reverse transcription kit (Primer Design, Southampton, UK). Quantitative PCR (qPCR) amplification was performed in 96-well plates in a mastermix for probes (Roche, Burgess Hill, UK) and run on a LightCycler^®^ 96 System (Roche). The qPCR amplifications for the human *AXL* (assay I.D. Hs.PT.56a.1942285), *MERTK* (assay I.D. Hs.PT.58.2640315), *TYRO3* (assay I.D. Hs.PT.58.38778546) and *GAS6* (assay I.D. Hs.PT.58.21535693) genes were performed using pre-designed primers/probes purchased from Integrated DNA Technologies (Leuven, Belgium). The amplification procedure entailed 45 cycles of 95°C for 10sec followed by 60°C for 30sec. Relative expression analysis was performed using the equation N = N_0_ x 2^Cp^ (LightCycler®96 software; Roche), normalising against the gene for ATP synthase subunit beta (*ATP5B*), which was determined in-house as the best internal reference gene out of 12 genes tested (geNorm kit, Primerdesign, Southampton, UK; data not shown).

### Immunohistochemistry

Brain tumour tissue array sections on glass slides, with normal tissue as control (GL805a; US Biomax, Rockville, MD) were deparaffinised using histology grade xylene (Sigma-Aldrich, Dorset, UK) for 5min and rehydrated using descending concentrations (100%, 70%, 50%, 30%) of molecular grade ethanol (Fisher Scientific) for 1min and 30sec respectively. Slides were then rinsed for 2min in water. Antigen unmasking was performed in 0.01 M sodium citrate (Fisher Scientific), pH 6 at 95°C with two changes for 5min each. The slides were washed twice for 2mins in deionized water. Endogenous peroxidase activity was blocked by incubating with 0.1% hydrogen peroxide (Sigma-Aldrich) at room temperature. Following three 5min washes with Phosphatase Buffered Saline (PBS), the slides were blocked with 1.5% rabbit serum in PBS for 1h. The test slide was then incubated with anti-Axl antibody at 1:100 dilution (goat polyclonal; R&D Systems) in 1.5% rabbit serum/PBS for 2h. The slides were then washed with PBS 3 times for 5min and incubated with biotinylated anti-goat IgG secondary antibody (Sigma-Aldrich) in 1.5% rabbit serum/PBS for 1h at room temperature. The slides were then incubated for 30 min with avidin-biotin enzyme reagent VECTASTAIN ABC Kit (Vector laboratories, Peterborough, UK) and washed with PBS three times for 5min. The slides were then developed using ImmPACT DAB Peroxidase (HRP) Substrate (Vector laboratories) for 10sec and washed with deionizing water three times for 5min. The tissues were counter-stained with haematoxylin and dehydrated through increasing concentrations of alcohols (50%, 70% 95% 100%) for 30sec and 1min respectively, followed by xylene for 1min, after which they were mounted with DPX (Sigma-Aldrich). Images were captured by a Zeiss Axiophot brightfield microscope at 20x magnification.

### Western blotting

Cells were briefly rinsed in ice-cold phosphate-buffered saline (PBS; Fisher Scientific) and lysed in ice-cold RIPA lysis buffer (150 mM NaCl, 1% Triton X-100, 0.5% sodium deoxycholate, 0.1% SDS, 50 mM Tris pH 8.0) supplemented with a cocktail of protease and phosphatase inhibitors (Fisher Scientific). Cell lysates were clarified by centrifugation and the proteins electrophoretically separated by 10% SDS polyacrylamide gel electrophoresis (SDS-PAGE). Cell lysates were also prepared from primary human brain microvascular endothelial cells (hCMEC/D3) and primary human cerebellar astrocytes (HA-c) (ScienCell, Carlsbad, CA). The separated proteins were transferred by a wet transfer method onto an activated polyvinylidene fluoride membrane (Millipore, Nottingham, UK). Membranes were incubated for 1h at room temperature in blocking buffer, which was either Tris-buffered saline-Tween 0.1% (TBS-T; Fisher Scientific) containing 3% non-fat dry milk, or otherwise containing 3% bovine serum albumin (BSA; Fisher Scientific) if probing for phosphorylated proteins. After blocking, membranes were incubated with primary antibody diluted in appropriate blocking buffer overnight at 4°C, then washed (3 × 5min) with TBS-T and incubated with appropriate horseradish peroxidase (HRP)-conjugated secondary antibody diluted in appropriate blocking buffer for 1h at room temperature. Following washing with TBS-T (3 × 5min), membranes were incubated with an enhanced chemiluminescence development reagent (Luminata Forte; Millipore) for 3min and visualised with a high sensitivity CCD camera imaging platform (Chemidoc MP; Bio-Rad, Hemel Hempstead, UK). The software *ImageJ* [36] was used for densitometric quantification of western blot band intensities.

The primary antibodies (and dilutions) used were: Axl (C-20) (goat polyclonal; 1:1,000), Tyro3 (C-20) (goat polyclonal; 1:1,000), MerTK (B-1) (mouse monoclonal; 1:1,000), p-EGFR and EGFR (goat polyclonal; 1:1,000) (Santa Cruz), p-Axl (rabbit polyclonal; 1:500; R&D systems), p-Akt 1/2/3 and Akt 1/2/3 (rabbit polyclonal; 1:1,000; Santa Cruz), p-Sky/Mer (rabbit polyclonal; 1:1,000), β-Actin (rabbit polyclonal; 1:5,000) (Sigma, Poole, UK). Secondary antibodies used were donkey anti-rabbit HRP (1:2,000; Dako, Cambridge, UK), anti-goat HRP (1:5,000) and anti-mouse HRP (1:5,000) (Promega, Southampton, UK).

### Cell survival/growth assay

1,500 cells per well were seeded in 96-well plates and incubated overnight, prior to various treatments (see above). Three or seven days post drug or Gas6 treatment respectively, 3-(4,5-dimethylthiazol-2-yl)-5-(3-carboxymethoxy-phenyl)-2-(4-sulfo-phenyl)-2H-tetrazolium (MTS) (Fisher Scientific) was added to cells at 0.4 μM in the presence of 0.3 nM phenazine methosulfate (Sigma) and incubated for a further 2h, after which absorbance was measured at 492 nm using a spectrophotometric microplate reader (Synergy; BioTek, Potton, UK).

### Apoptosis assay

30,000 cells per sample were treated with 2.5 μM and 5 μM (SNB-19) or 1 μM and 2 μM BGB324 (UP007) or vehicle (DMSO) for 24h. Following drug treatments the cells were centrifuged at 400 x *g* for 5min and resuspended in Annexin V binding buffer and incubated with Annexin V-CF488A conjugate and Hoechst 33342 (Insight Biotechnology, Wembley, UK) for 15min at 37°C using a heating block. The cells were then spun down at 400 x *g* for 5min and washed with Annexin V binding buffer twice by repeating the centrifugation and resuspension. Finally the cells were resuspended in 100 μl supplemented with 10 μg/ml propidium iodide (Insight Biotechnology) and 30 μl was loaded onto a special chamber slide (NC-Slide A2™) and cell populations were analysed for Annexin V/PI fluorescence (NucleoCounter® NC-3000™; Chemometec A/S, Allerød, Denmark) according to the manufacturer's protocol.

### Scratch wound assay

Linear cell migration along a surface was measured by scratch wound assay, where a linear scratch was made in a confluent cell monolayer with a 200 μl yellow pipette tip. Following injury, wound closure was monitored using an inverted Zeiss Axiovert 200M microscope housed in a live cell imaging chamber under normal cell culture conditions (37°C, 5% CO_2_, humid atmosphere). Images of 4 points per well were captured every 3h over a total period of 21h. Image analysis following the experiment was performed using *ImageJ*, and cell migration rates (area change/h) calculated thereafter.

### Cell motility assay

GBM cells were seeded in 24-well plates at low density (1,500 cells/well). Following treatment with BGB324 or vehicle (DMSO), live cell microscopic images of 4 points per well were taken every 20min for 24h under normal culture conditions (37°C, 5% CO_2_, humid atmosphere) using a Zeiss Axiovert 200M inverted live cell microscope with time-lapse imaging at 10x magnification. Quantification of cell tracking, measuring distance and trajectory were performed using *ImageJ* with its Cell Tracking plug-in. In total, 60 cells per condition were tracked randomly for a period of 3h, with total distance (μm) and average velocity (μm/min) being calculated.

### Colony growth assay

Wells of 24-well plates were first coated with 0.5 mL of complete medium/0.5% agar, and then GBM cells suspended in complete medium/0.35% agar were seeded at low density (1,500 cells/well). Cells were incubated for 2 weeks in total, with media and treatments being replenished weekly. Colonies present at the end of the incubation period were stained by incubation with 0.05% crystal violet solution (Fisher Scientific) for 1h. Images were taken of each well using a Zeiss Stemi SVG dissecting microscope (1.5 x magnification), and colonies were counted in 5 different fields of view.

### Cell invasion assay

Modified Boyden transwell chambers were used to assess cell invasion through extracellular matrix. Briefly, polycarbonate hanging inserts (Corning, Amsterdam, The Netherlands) were coated with 60 μg/ml Geltrex™ LDEV-free reduced growth factor basement membrane matrix (Life Technologies). Invasion in the cell culture incubator was allowed to occur over 6h for UP007 cells (20,000 cells/well) and 16h for SNB-19 cells (10,000 cells/well). Non-invading cells on the upper surface of the filter were removed with a cotton swab and cells that had invaded and adhered to the lower side of the filter were fixed for 15min with 37% formaldehyde solution containing 10-15% methanol (Sigma). Following fixation and brief wash with PBS, the adherent cells were stained with 0.5% crystal violet. Images were captured by a Zeiss Axiophot brightfield microscope and cells were counted in 5 random fields at a 10x magnification. Invasion was expressed as mean fold ± standard error of the mean (SEM) of the number of total cells counted per well.

### Statistical analyses

All data are expressed as mean±SEM, obtained from a minimum of 3 independent experiments, each constituting multiple replicates per condition. Quantitative data were analysed by Analysis of Variance (ANOVA) with *post-hoc* Bonferroni test for multiple comparisons with one control group “or multiple time points/treatments, or paired t-test for comparisons of control with treatment “ Statistical analyses and graphical representations were performed using Prism (GraphPad Software Inc). The level of statistical significance is indicated in the figures and accompanying legends. Western blot image processing was performed using Adobe Photoshop CC 2014 software (Adobe Systems Incorporated, CA, USA).

## SUPPLEMENTARY MATERIAL FIGURES


