# Correction: Small molecule inhibition of Axl receptor tyrosine kinase potently suppresses multiple malignant properties of glioma cells

**DOI:** 10.18632/oncotarget.28713

**Published:** 2025-04-04

**Authors:** Mikaella Vouri, Qian An, Matthew Birt, Geoffrey J. Pilkington, Sassan Hafizi

**Affiliations:** ^1^Institute of Biomedical and Biomolecular Science, School of Pharmacy and Biomedical Sciences, University of Portsmouth, Portsmouth, UK

**This article has been corrected:** The authors discovered a duplication error in Figure 4C, where the pAkt blot for SNB-19 cells was mistakenly duplicated in place of the pAkt blot for UP007 cells.

The authors have provided uncropped Western blots for pAkt and Axl for both cell lines and replaced the incorrect image with the correct one from the original experiments. The band densitometry results, derived from the true original blots, remain unchanged and accurately displayed in the graphs.

This inadvertent error does not compromise the validity or integrity of the study’s findings, and this correction does not affect the conclusions of the study. The corrected Figure 4C, produced from the original data, is shown below.

Original article: Oncotarget. 2015; 6:16183–16197. 16183-16197. https://doi.org/10.18632/oncotarget.3952

**Figure 4:**
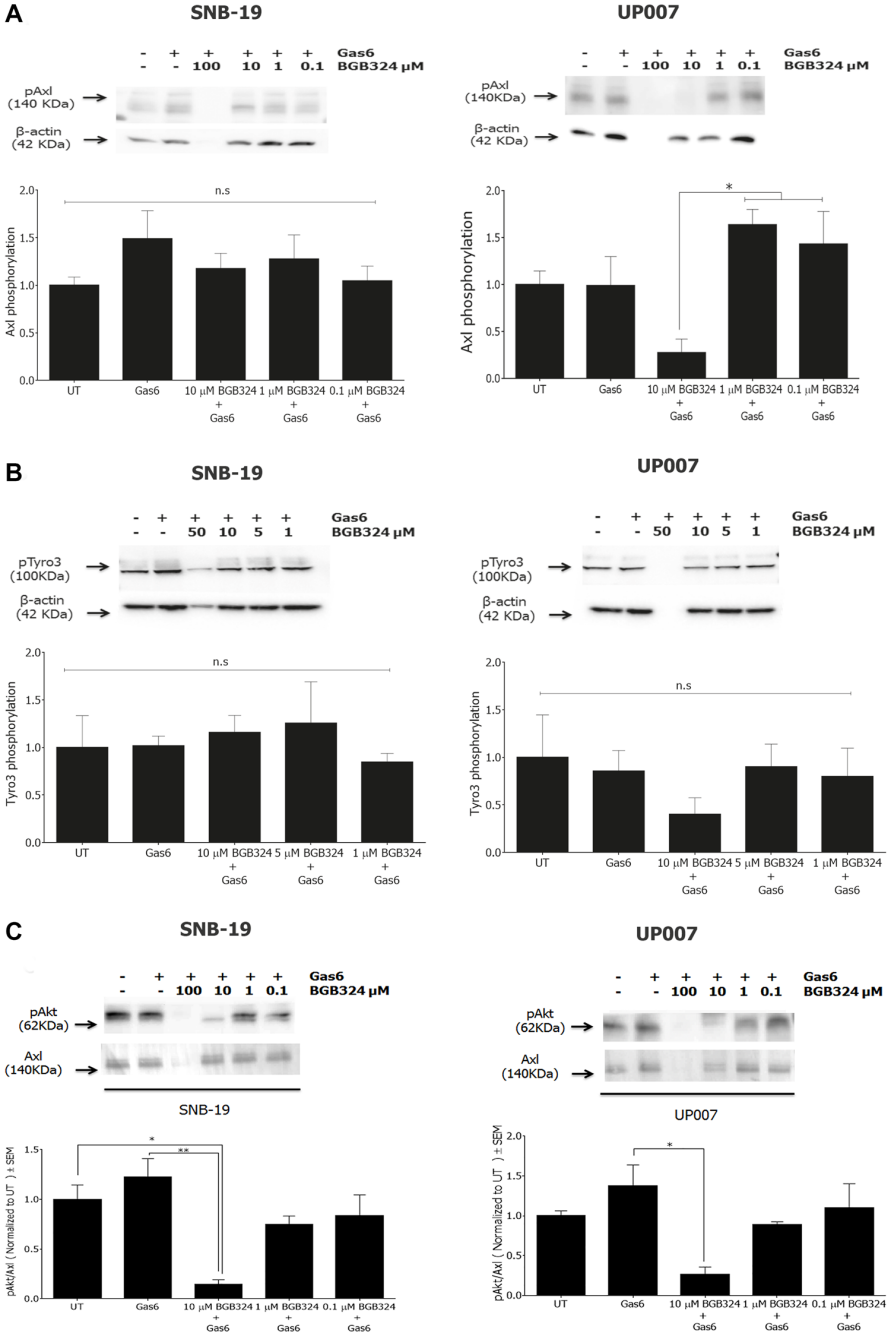
Comparative efficacies of BGB324 for inhibition of Axl, Tyro3 and Akt phosphorylation in GBM cells. Western blots showing inhibition by BGB324 (0.1–100 μM) of phosphorylation of Axl (**A**), Tyro3 (**B**) and Akt kinase downstream (**C**) stimulated by Gas6 (400 ng/ml) in SNB-19 and UP007 cells. Data are mean ± SEM (*n* = 3 separate experiments); ^**^ *p* < 0.01, ^*^ *p* < 0.05, ns, not significant, for comparisons indicated.

