# Supplementary material for: Small molecule inhibition of Axl receptor tyrosine kinase potently suppresses multiple malignant properties of glioma cells
Source: Oncotarget. 2015 Apr 29;6(18):16183–97. doi: 10.18632/oncotarget.3952 (PMC4599264; doi:10.18632/oncotarget.3952)
Supplement: Supplementary file 1 [file oncotarget-06-16183-s001.pdf]

Small molecule inhibition of Axl receptor tyrosine kinase potently suppresses multiple malignant properties of glioma cells

Supplementary Material

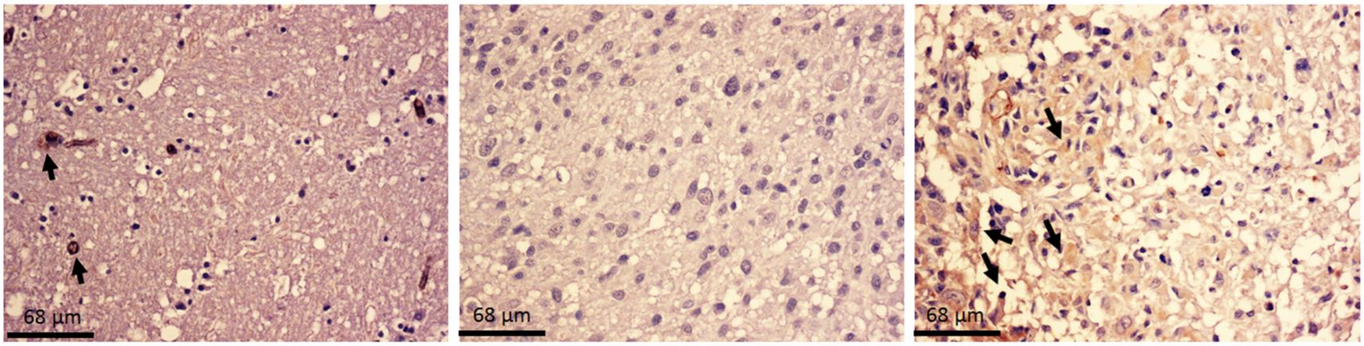

Supplementary figure 1. Images for Axl staining in normal brain (left image), Axl negative GBM (middle image) and Axl positive GBM (right image). Arrows represent Axl positive endothelial cells in normal brain vasculature and positive astrocytes in Axl positive GBM tissue.

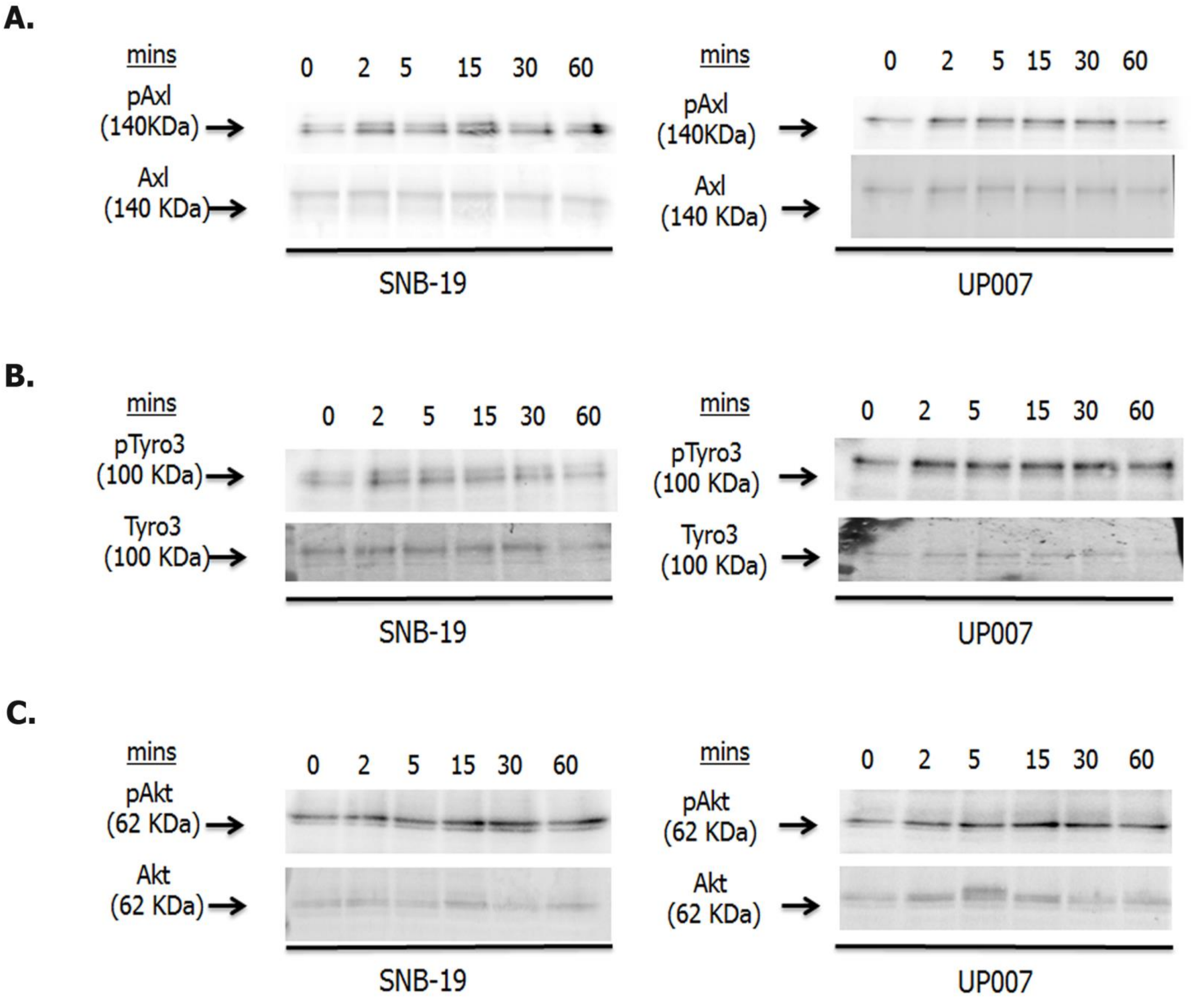

Supplementary figure 2. Western blot showing time-course of Axl phosphorylation by Gas6 (400 ng/ml) (A), of Tyro3 phosphorylation (B), and of pAkt levels (C) in SNB-19 and UP007 cells.

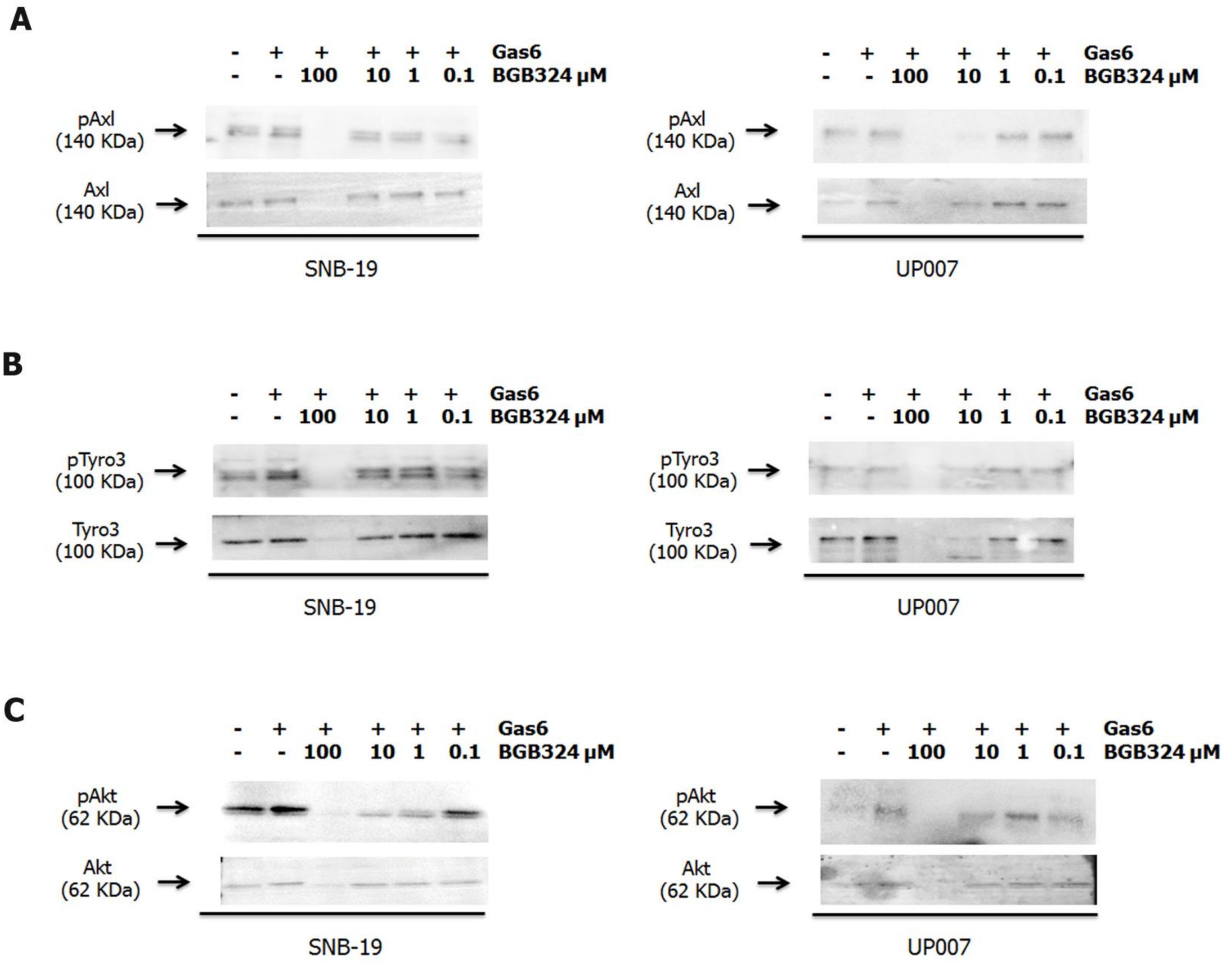

Supplementary figure 3. Western blots showing inhibition by BGB324 (0.1–100 μM) of phosphorylation of Axl (A), Tyro3 (B) and Akt kinase downstream (C) stimulated by Gas6 (400ng/ml) in SNB-19 and UP007 cells.
